# The association between foot-care self efficacy beliefs and actual foot-care behaviour in people with peripheral neuropathy: a cross-sectional study

**DOI:** 10.1186/1757-1146-2-3

**Published:** 2009-02-03

**Authors:** Byron M Perrin, Hal Swerissen, Craig Payne

**Affiliations:** 1 Faculty of Health Sciences, La Trobe University, Bundoora, 3086, Australia; 2 Musculoskeletal Research Centre, La Trobe University, Bundoora, 3086, Australia

## Abstract

**Background:**

People with diabetes and peripheral neuropathy often do not implement the foot-care behavioural strategies that are suggested by many health professionals. The concept of self-efficacy has been shown to be an effective predictor of behaviour in many areas of health. This study investigated the relationships between foot-care self-efficacy beliefs, self-reported foot-care behaviour and history of diabetes-related foot pathology in people with diabetes and loss of protective sensation in their feet.

**Methods:**

Ninety-six participants were included in this cross-sectional study undertaken in a regional city of Australia. All participants had diabetes and clinically diagnosed loss of protective sensation in their feet. The participants completed a self-report pen-paper questionnaire regarding foot-care self efficacy beliefs (the "Foot Care Confidence Scale") and two aspects of actual foot-care behaviour-preventative behaviour and potentially damaging behaviour. Pearson correlation coefficients were then calculated to determine the association between foot-care self-efficacy beliefs and actual reported foot-care behaviour. Multiple analysis of variance was undertaken to compare mean self-efficacy and behaviour subscale scores for those with a history of foot pathology, and those that did not.

**Results:**

A small positive correlation (r = 0.2, p = 0.05) was found between self-efficacy beliefs and preventative behaviour. There was no association between self-efficacy beliefs and potentially damaging behaviour. There was no difference in self-efficacy beliefs in people that had a history of foot pathology compared to those that did not.

**Conclusion:**

There is little association between foot-care self-efficacy beliefs and actual foot-care behaviour. The usefulness of measuring foot-care self-efficacy beliefs to assess actual self foot-care behaviour using currently available instruments is limited in people with diabetes and loss of protective sensation.

## Background

Foot pathology as a consequence of diabetes, such as foot ulceration leading to lower-limb amputation, is common and the global burden is set to increase with the world facing an epidemic of type 2 diabetes [1,2]. Individuals with a diabetes-related foot problem can use significantly more acute and community health services than individuals with diabetes without a foot problem [3,4]. Over 2,500 lower-limb amputations are performed each year in Australia and 80% of lower-limb amputations due to diabetes are thought to be preceded by a foot ulcer [5,6]. Furthermore, there are reports of high recurrence rates of foot problems such as ulceration even in established specialised foot clinics [7,8].

Previous research has identified which individual pathophysiological factors are involved in the precipitating events leading to lower limb amputation and its common precursor, ulceration. The "causal pathways" are invariably multi-factorial, with peripheral neuropathy being crucial in the development of both conditions [6,9-11]. In attempting to help people with diabetes and peripheral neuropathy these well understood pathophysiological pathways are often considered in isolation, but the outcomes of these predominately biochemical and biomechanical processes can be influenced by psychological, behavioural and environmental factors [12].

There is strong historical and anecdotal suggestion that certain foot-care behaviours can prevent diabetes-related foot pathology. However, the evidence suggests that people with diabetes often fail to employ the suggested behavioural strategies suggested in educational interventions. For example, two large population-based studies have found that only 20% of participants with diabetes inspected their feet daily and 23–25% never inspected their feet [13,14]. The wearing of appropriate protective footwear is also a significant issue. Two small cross-sectional studies from Europe found that in people at high risk of foot complications who were issued custom-made footwear to protect their feet only 22% wore their shoes all day and 53% most of the day respectively [15,16]. Armstrong and colleagues found that patients with neuropathic ulceration who were requested to wear a "removable walking boot" at all times found that the participants only wore the boot for an average of 28% of the total steps taken [17].

Patient education about appropriate self-care may have the potential to play a key role in preventing pathology, although a definitive randomised controlled trial to support its effectiveness is yet to be conducted. A recent high-quality review concluded that education appears to have a short-term positive impact on foot-care behaviour and may reduce the risk of foot ulcerations and amputations [18]. Education programs focus mainly on foot-care knowledge and behaviour and often emphasise concepts such as "foot care", "protection" and "foot inspection" [19-22]. These basic behavioural concepts are commonly included in patient education in an attempt to prevent foot-related problems, if inconsistently [23]. However, educational programs often fail to deal adequately with the psychological processes that are hypothesised to underlie self-care [24].

Understanding the factors that contribute to sub-optimal behavioural outcomes in foot-care is important if ulceration and amputation rates are to be decreased significantly. Behaviour has often been conceptualized as a function of environmental, personal and biological factors. Social cognitive theory is the best known and researched model for understanding the reciprocal relationship between these factors [25]. Fundamental to social cognitive theory is that individuals are proactively involved in their own development, adaptation and change [26]. In social cognitive theory, the extent to which individuals do so is mediated by self efficacy, the "...beliefs in one's capabilities to organise and execute the courses of action required to produce given attainments" [27]. How people behave for diverse purposes under diverse circumstances may be better predicted by the beliefs they have in the potential use of the skills they have [28]. Self-efficacy has been shown to be an explanatory framework in a wide range of health issues and has been an effective predictor of adherence to diabetes treatment regimes [29-32]. The theoretical construct of self-efficacy underpins this paper.

Two small cross-sectional studies have used self-efficacy as a framework to evaluate the self-efficacy beliefs, or "confidence" people with diabetes have in undertaking preventative foot-care behaviours [33,34]. In these studies, the participants were generally very confident they could undertake preventative foot-care behaviours. However, the high confidence levels found appear at odds with what is known about how people with diabetes actually behave toward their feet. It is incongruous that there are studies which demonstrate that people with diabetes have high levels of self-efficacy about undertaking preventative behaviours but other studies in fact suggest that there is a low prevalence of preventive foot-care actually being undertaken. Our review indicates that little research has been conducted on the association between foot-care self-efficacy beliefs, actual foot-care behaviour and foot pathology. The aim of this study was to investigate these relationships.

## Methods

Approval to undertake this cross-sectional study was obtained from the Human Ethics Committee of La Trobe University and the Human Research Ethics Committee of Bendigo Health. The study was carried out within a multidisciplinary "Diabetic Foot Clinic" in a regional city of Australia with a population of approximately 90,000 people, 90% of whom are Australian born [35]. People who had been admitted to the Diabetic Foot Clinic at any time from the years 2001 to 2007 were invited to participate.

Key inclusion criteria were a self-reported diagnosis of diabetes and clinically determined "loss of protective sensation" in the feet. The presence of loss of protective sensation was determined clinically by the principal researcher and defined as an inability to detect the 10 g Semmes Weinstein monofilament (Bailey, UK) on four or more sites on at least one foot, and/or a vibratory perception threshold of >25 V on at least one foot (Biomedical Instrument Co, Newbury, Ohio) [36]. The use of these neuropathy testing instruments in this way has been shown to be 100% sensitive and 77% specific in identifying people at risk of future neuropathic foot ulceration [36].

Exclusion criteria included an inability to understand English sufficiently to complete the self-report measures or an inability to sign the informed consent form. Participants provided informed consent before being interviewed and were assessed by the principal researcher to determine the following variables: age, gender, diabetes type, duration of diabetes, education, living arrangements and history of a diabetes-related foot pathology (ulcer, Charcot arthropathy, infection requiring admission to hospital, surgery and amputation). Previous medical records were consulted to confirm details of previous foot pathology.

**Figure 1:**
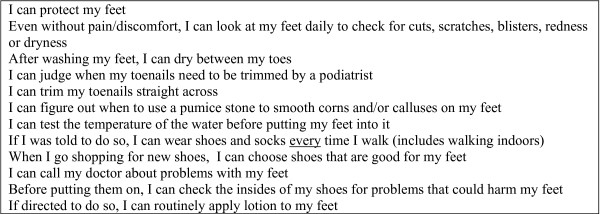
**The FCCS statements**.

To measure foot-care self-efficacy beliefs each participant completed the self-report "Foot Care Confidence Scale" (FCCS) questionnaire [34]. The development of the FCCS was guided by self-efficacy theory and was designed to combine the three dimensions of self-efficacy: magnitude, strength and generality. The FCCS consists of twelve statements (Figure 1) about the "confidence" people have in undertaking various foot-care activities using a five-point Likert scale response. In response to a statement about undertaking foot-care behaviour (e.g. "I can protect my feet"), the participant could respond with the following likert responses: "strongly not confident", "moderately not confident", "confident", "moderately confident" and "strongly confident". The FCCS has been shown to be internally consistent (Cronbach's α = 0.92), has a strong nursing content validity and has a one-dimensional construct; however criterion validity has yet to be demonstrated [34]. A maximum score of sixty is possible, with higher scores indicating a higher level of self-efficacy beliefs.

**Figure 2:**
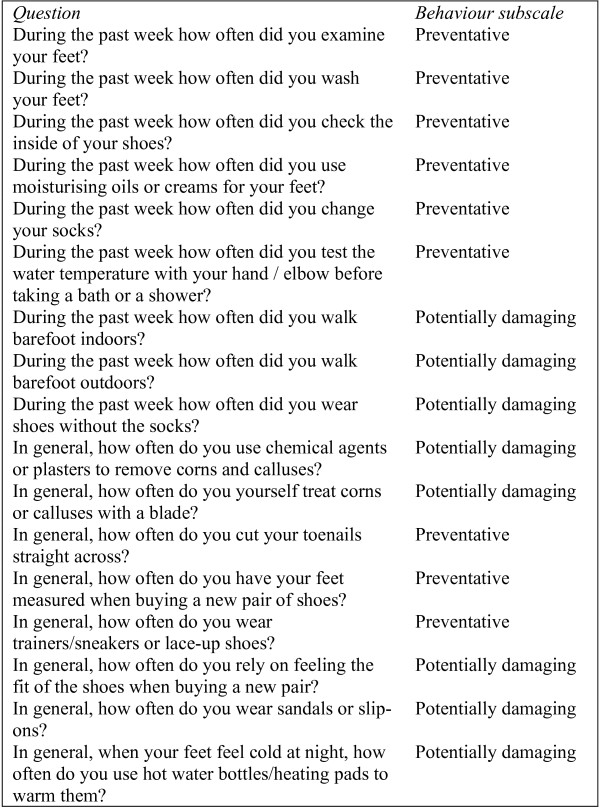
**The behaviour questions**.

To measure foot-care behaviour, a self-report questionnaire was used that was derived from a tool developed by Vileikyte and colleagues [24]. The content of the tool was based on international "diabetic foot care guidelines" [37,38]. However, a stringent validation process has yet to be published. The seventeen item questionnaire is split into two behavioural subscales: nine items pertaining to preventative behaviour and eight items to potentially damaging behaviour (Figure 2). Responses were rated on two different scales: a 6-point scale for "during the past week" questions (twice a day, daily, every other day, twice a week, once a week, or never) and on a four point scale for "in general" questions (always, most of the time, occasionally, or never). Because of the difference in scaling, items were converted to a scale that ranged from 0 to1 before summating scores. After re-coding, higher scores (i.e. closer to 1) indicated both more preventative and potentially foot-damaging behaviours [24].

SPSS 14.0 was used for the analyses. To determine the relationship of FCCS scores with both preventative and potentially damaging behaviour scores the Pearson product-moment coefficient of correlation was determined in each case. For these correlation analyses, an α < 0.05 was considered significant. A multiple analysis of variance test (with Bonferroni-type adjustment) was used to compare mean FCCS scores and behaviour subscale scores for participants that had a history of diabetes-related pathology with those that did not. Due to the Bonferroni-type adjustment, an α < 0.017 was considered significant for this test.

## Results

Characteristics of the participants are reported in Table 1. A high percentage of the participants were male and were diagnosed with type 2 diabetes, and there were a high proportion of participants that did not complete secondary school. There were a high proportion of participants who had a history of foot pathology.

**Table 1:** Characteristics of study population

*Variable*	*n *= *96*
Male sex (%)	76

Age (years)	64.49 (± 10.73)

Diabetes type (type 2%)	90

Diabetes duration (years)	15.89 (± 11.54)

Education (%)	
Completed primary school	6.3
Undertook some secondary school	80.1
Completed secondary school*	6.3
Undertook some university	2.1
Completed undergraduate university degree	3.1
Completed postgraduate university degree	2.1

Living arrangement (%)	
Spouse/partner/friends	72
Alone	28

History of foot pathology (%)	70

FCCS score	41.13 (± 10.71)

Preventative behaviour score	0.57 (± 0.14)

Potentially damaging behaviour score	0.20 (± 0.09)

Data are means (± standard deviations), unless otherwise stated

*In Australia, secondary school is generally completed at around 18 years of age, after 12 years of schooling.

Pearson product-moment coefficient of correlation was performed and showed a small positive correlation between FCCS scores and preventative behaviour scores (r = 0.2, p = 0.05) and virtually no correlation at all between FCCS scores and potentially damaging behaviours (r = -0.05, p = 0.61).

Multiple analysis of variance was performed to investigate differences in mean FCCS and behaviour subscale scores for those with a history of foot pathology and those without. When considering the FCCS and behaviour subscale scores separately, there was virtually no difference between the mean FCCS scores for those with a history of pathology compared with those with no history of pathology (F = 0.05, p = 0.95). For the preventative behaviour subscale, those that had a history of pathology reported undertaking more preventative behaviours, however this did not reach statistical significance after the Bonferroni-type adjustment (F = 5.62, p = 0.02). Although, those that had a history of foot pathology did report undertaking fewer potentially damaging behaviours than those without a history of foot pathology (F = 14.00, p =< 0.001).

## Discussion

To the authors' knowledge, this is the first study to have directly investigated the relationship between foot-care self-efficacy beliefs and actual foot-care behaviour. This study also investigated the relationship between foot-care self-efficacy beliefs and behaviour with history of foot pathology.

This sample has a preponderance of older males with type 2 diabetes that have low levels of education. These demographics are consistent with other populations of people with diabetes, peripheral neuropathy and who are at high risk of future diabetes-related foot problems [39-42]. These characteristics may influence the results of this study. For example, older people have been shown to have difficulty in undertaking basic foot-care behaviours such as inspection and appropriate nail care [43]. Males generally are less likely to rest or seek medical advice during an illness and engage in fewer health promoting activities [44]. Interestingly, with respect to diabetes care, males have been shown to have higher self-efficacy beliefs in managing their diabetes than females [45].

Surprisingly, self-efficacy was not a strong predictor of behaviour or pathology. With respect to behaviour, we found only a weak relationship between FCCS and preventative behaviour scores. While this indicates that the participants who have stronger foot-care self-efficacy beliefs also undertook more preventative behaviours the relationship was small, and unconvincing in its "clinical" significance in practice – even if statistically the result was significant. Furthermore, there was no association between foot-care self-efficacy beliefs and potentially damaging behaviour. While the FCCS only focuses on preventative behaviours, if there was a strong relationship between self-efficacy beliefs and behaviour it would be expected that those with weaker foot-care self-efficacy beliefs would be more likely to undertake potentially damaging behaviours. This was not found here, again suggesting that self-efficacy is not a useful predictive variable for foot-care behaviour.

These findings explain the apparent inconsistency in the literature that people with diabetes score highly on their self-efficacy for conducting appropriate foot-care and yet display low levels of actual preventive behaviour (as discussed above). It is likely that a more detailed analysis of the environmental and psychological variables related to actual behaviour will need to be conducted to establish the conditions that lead to higher levels of preventive behaviour. At least in this study, self-efficacy is not a sufficient predictor of foot-care behaviour. Neither was self-efficacy related to foot pathology outcomes. There was no difference in self-efficacy beliefs between those with a history of foot pathology and those with no history. As with the findings for lack of a relationship between self-efficacy and preventive and damaging behaviours, this result suggests that self-efficacy as measured by the FCCS has limited clinical utility for people with diabetes and loss of protective sensation in their feet.

Interestingly, the participants with a history of diabetes-related foot pathology did indicate that they undertook fewer potentially damaging behaviours. It would be expected that the participants who had suffered a serious problem of a diabetes-related foot problem would undergo more appropriate foot-care behaviours in the future such as avoiding potentially damaging behaviour. For these people, adverse outcomes may act as a prompt for preventive behaviour. Unfortunately, it appears that this "prompt" is required first before preventative behaviours are taken place, rather than implementing the preventative behaviours before they get a foot problem. Furthermore, the reported extremely high annual recurrence rate of diabetes-related foot pathology attests to the difficulty in preventing foot pathology despite any actual foot-care behaviour undertaken [7,8].

The results of this study need to be interpreted in the context of some limitations. The cross-sectional nature of the design ensured that the sample was only investigated at one point in time rather than exploring patterns of change over time, such as the changes in self-efficacy beliefs, actual behaviour or the development of a foot pathology [46]. Additionally, the FCCS and the self-reported behaviour scales were self-report inventories and are prone to response bias, particularly in this current sample of people with loss of protective sensation. For example, a study of health-seeking behaviour in people at high risk of diabetes-related foot pathology concluded that "...what subjects report they would do in hypothetical situations is not what occurs in reality" [47]. In addition, even though this study measured foot-care self-efficacy beliefs, Stuart and Wiles seriously doubt the worth of using quantitative tools to assess foot-care "knowledge" in people with diabetes as they found that their participant's actual understanding of foot-care practices derived from in-depth qualitative techniques fell well short of their apparent knowledge as investigated using quantitative techniques [48]. Furthermore, the manner in which actual behaviour was measured may be further flawed by the self-report inventory design. A combination of designs that include more in depth interviewing and actual observations may be more appropriate to measure actual behaviour. This would allow for a better understanding of the relationships between antecedents, behaviours and consequences and it may allow for specific behaviours to be targeted and recorded in a meaningful way in a natural setting [49].

Finally, it is important to be aware of the issue of sample size and its effect on tests of significance in relation to correlations we determined in our study. As the sample size was relatively large, we have taken a more conservative approach that focused on the correlation coefficient, which was poor. Although the correlation between FCCS scores and preventative behaviour scores was just statistically significant (p = 0.05), the correlation was actually poor (r = 0.02). A larger sample size would have ensured more statistical power, although the clinical implications of this are unknown.

## Conclusion

The management of people with diabetes-related foot problems must take place in a context that includes consideration of psychosocial and behavioural factors in addition to pathophysiological factors. However, this study has found that it is unlikely that the evaluation of foot-care self-efficacy beliefs is particularly useful in assessing the actual foot-care behaviour of people with diabetes and loss of protective sensation in their feet. Detailed prospective research is now required to definitively determine the relationship between self-efficacy beliefs and the incidence of diabetes-related foot pathology.

## Competing interests

The authors declare that they have no competing interests.

## Authors' contributions

BMP undertook the conception, design, data collection, statistical analysis and preparation of the manuscript. HS participated in the design, statistical analysis and participated in the drafting of the manuscript. CP provided general support and supervision and participated in the drafting of the manuscript. All authors read and approved the final manuscript.
